# Cholesterol metabolism-related genes predict immune infiltration and prognosis in gastric cancer patients

**DOI:** 10.7150/jca.104389

**Published:** 2025-03-10

**Authors:** Wenxuan Liu, Li Liu, Tianrui Kuang, Wenhong Deng

**Affiliations:** Department of General Surgery, Renmin Hospital of Wuhan University, Wuhan, Hubei Province, China.

**Keywords:** gastric cancer, cholesterol metabolism, immune, prognosis, immunotherapy treatment

## Abstract

**Background:** Gastric cancer (GC) is one of the most prevalent malignant diseases worldwide. Abnormal metabolic reprogramming, particularly cholesterol metabolism, influences tumor development and treatment outcomes. This study investigates the predictive and functional significance of cholesterol metabolism-related genes in gastric cancer patients.

**Methods:** Clinical and gene expression data related to cholesterol metabolism in gastric cancer were analyzed using datasets from the Gene Expression Omnibus (GEO) and The Cancer Genome Atlas (TCGA). A predictive signature was developed and validated using LASSO, Cox regression, and the GSE26889 cohort, followed by evaluation with Kaplan-Meier analysis. A nomogram was constructed by integrating the signature with clinical factors and ssGSEA for immunological analysis. The role of NPC2 was investigated using western blot, qPCR, and cellular assays.

**Results:** We conducted a bioinformatics analysis of 50 genes associated with cholesterol metabolism in gastric cancer. Using the GEO and TCGA datasets, we identified 28 genes with differential expression in gastric cancer patients. Subsequent COX univariate and LASSO regression analyses of these 28 DEGs identified five genes (APOA1, APOC3, NPC2, CD36, and ABCA1) as independent prognostic risk factors. We then constructed a risk model for cholesterol metabolism genes, revealing that survival was worse in the high-risk group compared to the low-risk group, with more severe case staging outcomes. We conducted a comparative analysis of immune cells between the high-risk and low-risk groups, revealing distinct variations in immune cell type expression. We then developed a model using a correlation nomogram to illustrate these conclusions. We further examined the biological characteristics of NPC2. Immunohistochemistry and qPCR results showed that NPC2 exhibited significant protein and mRNA expression in gastric cancer tissues. We used siRNA technology to suppress NPC2, resulting in reduced viability, proliferation, and invasion capacity of gastric cancer cells, as determined by CCK-8, colony formation, wound healing, and Transwell assays.

**Conclusion:** A risk signature comprising five cholesterol metabolism-related genes was constructed using bioinformatics to estimate outcomes and therapeutic responses in gastric cancer patients. The results suggest that NPC2 may serve as a novel biomarker for gastric cancer patients.

## Introduction

Gastric cancer (GC) is a significant disease on a global scale. GC is the fifth most commonly diagnosed carcinoma globally, with approximately one million new cases each year. Due to its typically late-stage diagnosis, gastric cancer has a high mortality rate and ranks third among cancer-related deaths 1. Development of gastric cancer is a multistage, gradually progressing, and complex pathological process 2. Pylori infection, age, obesity, smoking, alcohol use, and family history of disease are all risk factors for gastric cancer 3. Additionally, aberrant molecular signalling pathways, epigenetic changes, and genetic abnormalities all contribute to the growth, metastasis, and spread of gastric cancer 4. Gastric cancer treatment has progressed considerably over the last few decades, with systemic chemotherapy, radiotherapy, surgery, immunotherapy and targeted therapy all proving effective in gastric cancer 5. However, the incidence and mortality rates of GC remain high. Therefore, it is crucial to identify early diagnostic and prognostic biomarkers to improve gastric cancer outcomes.

Cholesterol is crucial for maintaining the integrity and function of cell membranes. Abnormal cholesterol metabolism is intricately linked to tumor development and progression 6. The role of cholesterol metabolism in the development and progression of various cancers, including gastric cancer, is becoming increasingly recognized 7. Cancer cells typically exhibit altered cholesterol metabolism, which provides the necessary support for their rapid growth and proliferation 8. Similarly, increased synthesis of new cholesterol or uptake of exogenous cholesterol can also promote rapid growth and tumor formation 9. Cholesterol-rich lipid rafts in cell membranes play a significant role in signal transduction pathways that regulate cell proliferation, survival, and migration. The aberrant activation of these pathways has been identified as a contributing factor in the development of gastric cancer 10. A number of clinical studies have sought to elucidate the relationship between cholesterol levels and the prognosis of gastric cancer. Although the results are inconclusive, some studies indicate that cholesterol control may improve prognosis and reduce the risk of recurrence 11. Furthermore, cholesterol metabolism has been shown to induce drug resistance and immune escape in gastric cancer cells by remodeling the tumor microenvironment 12 13 14 15. Cholesterol is a vital component for the healthy development of cells, playing a pivotal role in the formation of eukaryotic cell membranes 16. Its functions are instrumental in determining the profound impact it exerts on cells within the body 17. Abnormal cholesterol metabolism has recently been identified as one of the most important metabolic indicators of tumor cells 18.

This research scrutinized the manifestation and plausible function of genes implicated in cholesterol metabolism within gastric carcinoma. The prognostic model was formulated within the TGCA repository and authenticated through the GEO dataset. A sequence of bioinformatic assessments revealed that the cohort categorized as high-risk exhibited a more unfavorable prognosis and was correlated with a more adverse clinicopathological stage. Our investigation has introduced an innovative risk prognostication model for cholesterol metabolism and verified the biological conduct of NPC2, a pivotal gene in cholesterol metabolism, thereby offering fresh modalities and viewpoints for gastric cancer treatment.

## Materials and Methods

### Data Acquisition

On 22 August 2022, raw RNA sequencing data for gastric cancer were collected from the Tumor Genome Atlas (TCGA) database (https://www.cancer.gov/about-nci/organization/ccg/research/structural-genomics/tcga). The GSE26899 dataset was extracted from the Gene Expression Omnibus (GEO) database (https://www.ncbi.nlm.nih.gov/geo/) and included gene expression levels and clinically relevant features.

### Cholesterol-related differentially expressed genes

Finding 50 cholesterol metabolism genes in the GEO and TCGA databases after extracting cholesterol metabolism-related genes from the cholesterol pathway (Table S1). We examined the differential expression of genes concerned with fatty acid metabolism in samples of healthy and tumor using the "limma" R program. Depending on false discovery rate (FDR) <0.05 and | log2 FC| >1, the 28 genes associated to cholesterol metabolism that were differently expressed between controls and GC patients were identified.

### Building and testing a prognostic signature for genes associated to cholesterol

The validation group was composed of GEO samples (GSE26899), whereas the training group was made up of TCGA samples. Through sample ID, the expression levels of the genes engaged in cholesterol metabolism that are differently expressed for each sample were coupled with the appropriate prognostic outcomes. By using univariate Cox regression analysis on the training set, genes with p values < 0.05 were chosen from the differential genes linked to cholesterol metabolism that were examined for prognostic factors. GC samples from the training set were examined for mutations and correlations using the "maftools" R program. The "glmnet" R tool was utilized to further evaluate genes linked to prognosis, and Cox regression analysis together with the least absolute shrinkage and selection operator (LASSO) were employed to construct a prognostic risk signature.

The calculation formula is shown in the previous study 19. Principal component analysis (PCA) was performed using the R programs "limma" and "ggplot2", respectively, on genes related to cholesterol and genes used to create prognostic models. In accordance with the median of the risk scores, the samples were severed into two groups: low risk score groups and high risk score groups. The distinction in overall survival (OS) between the groups with low and high risk scores was compared using Kaplan-Meier analysis and the log-rank test. The "survivalROC" R program was utilised to create time-dependent ROC curves to assess the prognostic risk score model's prediction power. Eventually, the test set was used to further confirm the prognostic risk score model's accuracy and relevance.

### Clinical characteristics and risk scores in relation to each other

In the TCGA cohort, according to the sample ID, the risk score from every sample was combined with the appropriate clinical traits. Using the "limma" R package, the connection among risk scores and clinical features (such as pathological classification, gender, pathological stage, and AJCC TNM stages) was investigated.

### Nomogram creation for OS prediction

Based on the TCGA cohort, a nomogram containing age, sex, pathological stage, pathological categorization, and prognostic risk score was created in R using the "rms" package in order to forecast the OS of GC patients. Time-dependent calibration curves were formed to determine the exactness of the nomogram in forecasting the survival of people with GC at 1, 3, and 5 years. In addition, ROC curves were designed to calculate the AUC to evaluate the nomogram's and the prognostic risk score model's prediction accuracy.

### Immunotherapy analysis in the high and low score groups

For every sample in the TCGA cohort, the degree of immune-related infiltration was calculated using ssGSEA utilizing the "GSEABase" R packages and "GSVA" R packages. These gene sets were gathered in previous studies to assess immune-related features in TME, including a wide range of distinct subtypes of human immune cells and immunological-related activities. Between the low risk group and the high risk group, differences in enrichment scores were examined. The associations between immune cells and genes related to prognosis were also examined. Finally, the TIDE (http://tide.dfci.harvard.edu/) algorithm was utilised to estimate the feasibility of immunotherapy for low and high risk subgroups 20.

### Expression of gene RNA and protein

Immunohistochemistry (IHC) analyzed protein expression in tumor tissues using anti-NPC2, ABCA1, APOC3, APOA1, and CD36 antibodies. DAB solution marked positive signals, and hematoxylin visualized nuclei. Images were obtained with an optical microscope (Olympus, Japan).

RNA isolation and RT-PCR assessed gene expression in tumor tissues. TRIzol reagent (Invitrogen, USA) extracted RNA, PrimeScript RT kit (Takara, Japan) synthesized cDNA, and qRT-PCR was performed with SYBR Green II Mixture kit (Takara, Japan) on the ABI StepOne system (Applied Biosystems, USA). β-tubulin normalized mRNA expression using the 2-ΔΔCt method. Primers were from Sangon Biotech (Shanghai).

APOA1-F: 5'-AAGTGGCAGGAGGAGATGGAG-3', APOA1-R:5'-CAGTGGGCTCAGCTTCTCTTG-3', β-tubulin-F: 5'GCAATAGCACAGCCAGGAGGAG-3', β-tubulin-R: 5'-TCAGCCTCGGTGAACTCCATCTC-3', APOC3-F: 5'-TACATGAAGCACGCCACCAAG-3', APOC3-R: 5'-TACATGAAGCACGCCACCAAG-3', NPC2-F: 5'-AGGACTGCGGTTCTGTGGATG-3', NPC2-R: 5'-TTGCTGGTGAAGGTGACATTGAC-3', ABCA1-F: 5'-GTGGTGTTCTTCCTCATTACTGTTC-3', ABCA1-R: 5'-CCTCACATCTTCATCTTCATCATTCAG-3', CD36-F: 5'-AACCTATAACTGGATTCACTTTACAATTTG-3', CD36-R: 5'-GGCACAATATAGTTCCTCTTCAGATTC-3'.

### Cell culture and transfection

RPMI-1640 media (HyClone, Logan, UT, USA) supplemented with 10% fetal bovine serum (FBS; BI, Kibbutz, Israel) was used to cultivate the human gastric cancer cell lines AGS and HGC-27, which were obtained from ATCC or Shanghai Cell Bank (Shanghai, China). The culture was conducted at 37°C in a 5% CO2 atmosphere. For the NPC2 knockdown experiment, NPC2-specific siRNA was designed and purchased from GenePharma (Shanghai, China). AGS and HGC-27 cells were uniformly seeded one day before transfection, followed by transfection using transfection reagent (Thermo Scientific, R0532, Waltham, USA) small interfering RNA (siRNA) targeting NPC2 (sense: AGGACTGCGGTTCTGTGGATG, antisense: TTGCTGGTGAAGGTGACATTGAC) for 48 hours.

### Cell proliferation assay

Prepare a cellular suspension, quantify it, and seed 100μl per well into a 96-well plate. Subsequently, place the plate within the incubator for a prescribed duration to foster cellular adherence to the vessel walls (approximately 2-4 hours). Treat the cells in accordance with the experimental requisites, encompassing medications, chemicals, gene manipulations, and more. Ensure a minimum of 3 replicas for each experimental cohort alongside a designated control group. Following this, continue incubating the plates under specified conditions, tailoring the incubation period to the cells' sensitivity towards experimental variables. Introduce 10μl of CCK-8 solution into each well, gently agitating the culture plate for fusion. Incubate the plates within the incubator for a span of 1-4 hours. Given the varying oxidative activities of diverse cell types and the consequent metabolite generation, the suitable incubation duration can be deduced through preliminary trials. Subsequently, ascertain the absorbance at 450 nm for both the experimental and control clusters utilizing an enzymatic label.

### Wound healing and Transwell assay

In 6-well plates, transfected cells were planted. Once cell density reached 90%, a 200-µl sterile pipette tip was used to make a scratch. Photographs were taken after 24 hours of cultivation in serum-free media using an inverted microscope. To examine cell invasiveness, Matrigel-precoated Transwell chambers were used. Serum-containing media was placed in the lower chamber, and serum-free medium with transfected cells in the upper chamber. After 24 hours at 37°C, cells were fixed, stained with crystal violet, and counted under a microscope.

### Statistical analysis

Data were analyzed using R 4.2.1 and GraphPad Prism 8. T-test was used for differences between the two groups. Kaplan-Meier methods and log-rank test were applied to assess survival outcomes. Associations were evaluated using Spearman's correlation analysis. P< 0.05 was considered the borderline value for statistical significance (p > 0.05, *: p ≤ 0.05, **: p ≤ 0.01, ***: p ≤ 0.001).

## Results

### Identification of differentially expressed cholesterol metabolism-related gene

**Figure 1:**
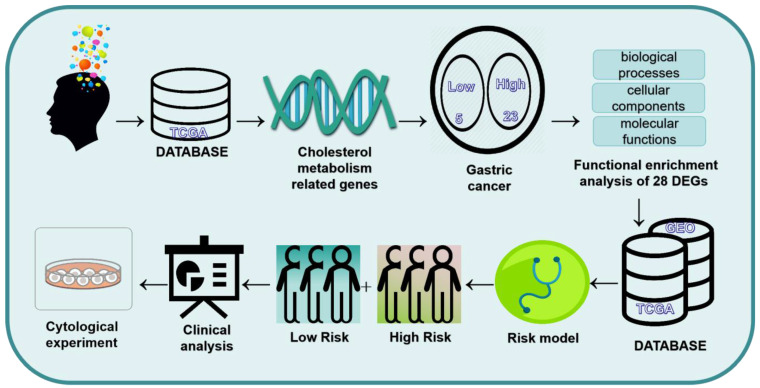
The flowchart of the study.

**Figure 2:**
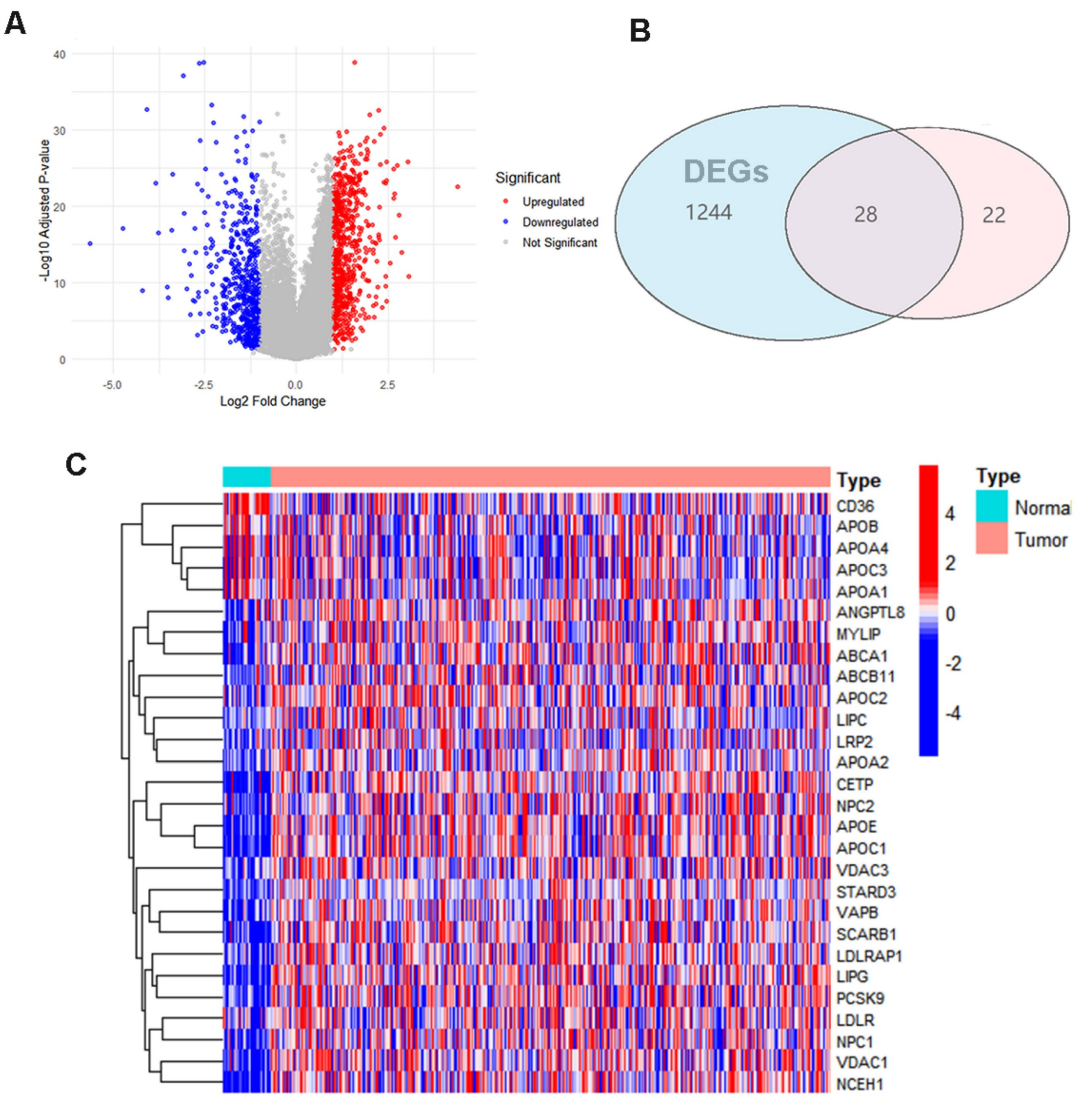
Cholesterol metabolism-related differential genes select: **(A)** Differential gene volcano plot of TCGA gastric cancer patients **(B)** Differential genes and cholesterol metabolism gene Venn diagrams **(C)** Heatmap of cholesterol metabolism-related differential genes.

The flowchart of this study is shown in Figure 1. 1272 Differences in gene expression in normal tissue (n = 32) and gastric cancer tissue (n = 371) samples were gathered from TCGA dataset (Fig. 2A). Combining the differential genes and cholesterol metabolism genes, the Venn diagram showed 28 cholesterol metabolism-related differential genes (Fig. 2B). In the tumor samples, the heat map shows the 5 genes were lowly expressed and 23 genes were highly expressed (Fig. 2C).

### Functional enrichment analysis of differential genes

**Figure 3:**
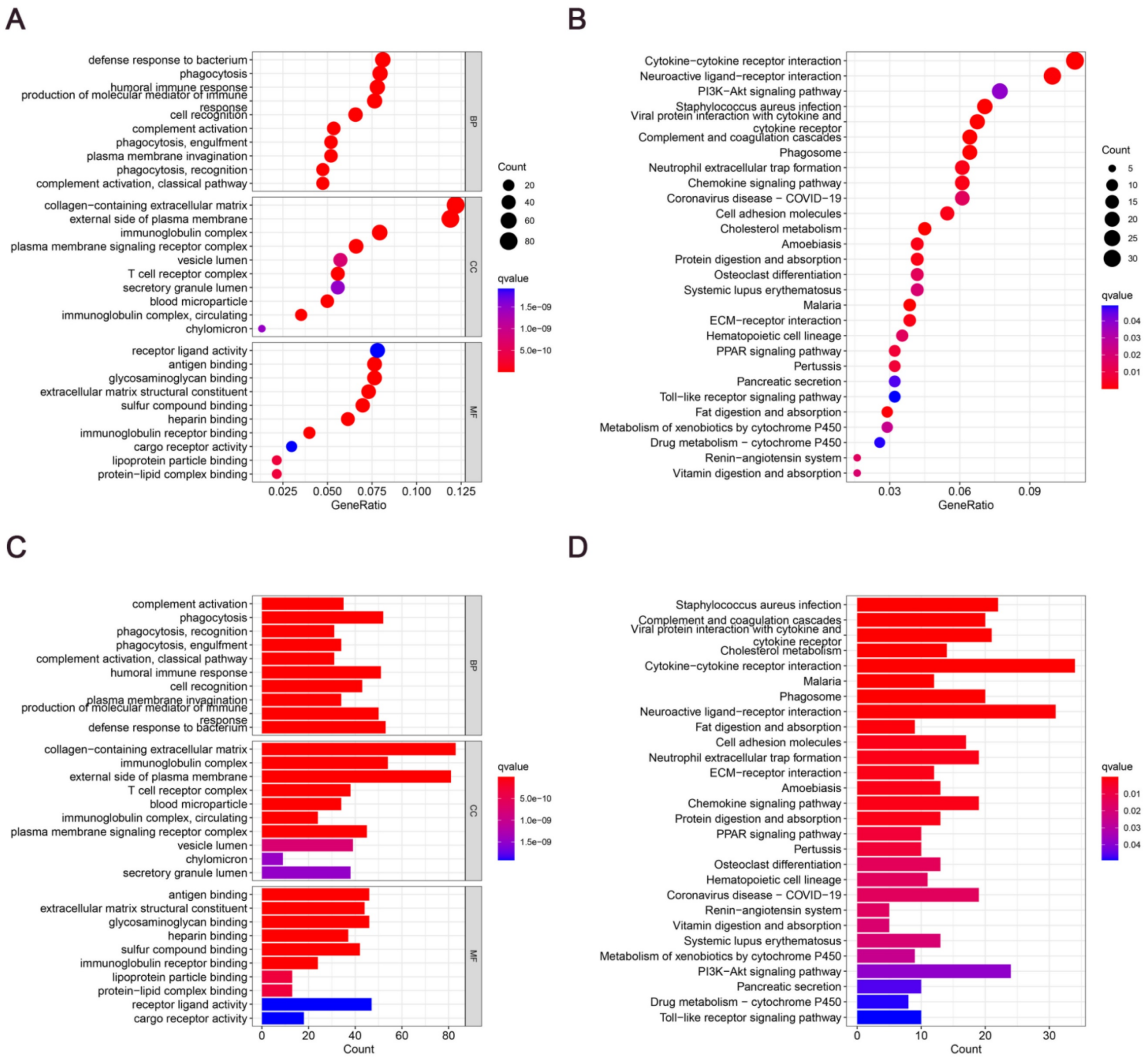
Enrichment analysis of differentially expressed genes involved in cholesterol metabolism: **(A and C)** Dot plot and bar graph showing GO analysis. **(B and D)** Dot plot and bar graph showing KEGG analysis.

To elucidate the potential mechanisms of cholesterol differentially expressed genes, functional enrichment analysis was done on 28 DEGs. In the biological processes, the DEGs were primarily abundant in defense response to bacterium, phagocytosis, humoral immune response, cell recognition, complement activation. In the cellular components, the DEGs were primarily abundant in collagen-containing extracellular matrix, external side of plasma membrane, immunoglobulin complex, plasma membrane signaling receptor complex. In the molecular functions, the DEGs were primarily abundant in antigen binding, glycosaminoglycan binding, extracellular matrix structural constituent, lipoprotein particle binding, protein-lipid complex binding (Fig. 3A and 3C). In the KEGG pathways, the DEGs were primarily abundant in Cytokine-cytokine receptor interaction, Phagosome, PPAR signaling pathway, Fat digestion and absorption (Fig. 3B and 3D). These findings imply that GC development depends significantly on cholesterol metabolism.

### Building a prognostic risk signature

**Figure 4:**
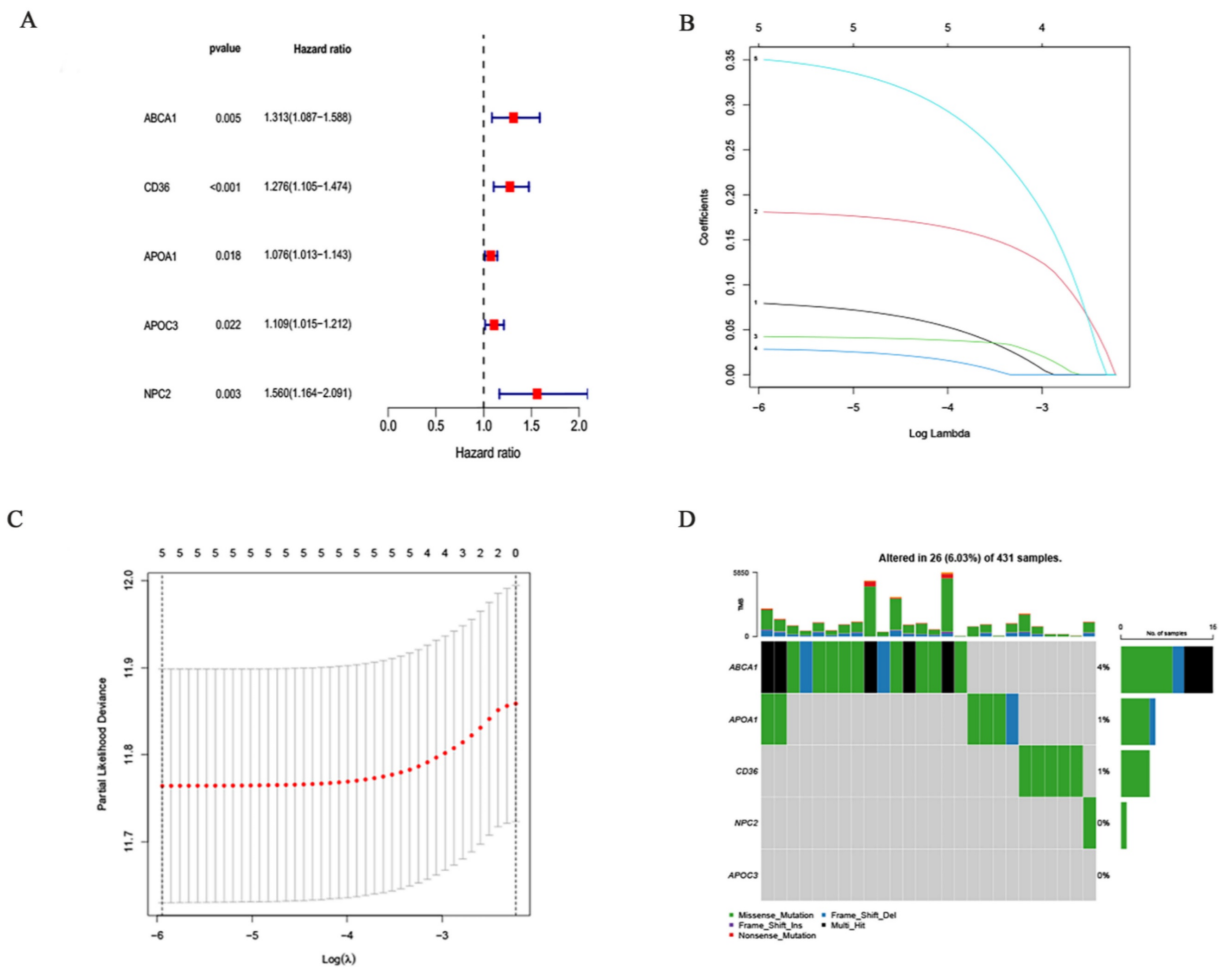
Constructing Prognostic Risk Score Model: **(A)** Five DEGs significantly linked to OS identified by univariate Cox regression. **(B, C)** Five prognostic DEGs selected using LASSO regression with ten-fold cross-validation. **(D)** Mutation frequencies of the five cholesterol metabolism-related genes in GC patients.

The training cohort consists of samples from the TCGA dataset, and the validation cohort consists of samples from the GEO dataset. On 28 differentially expressed cholesterol metabolism-related genes, an examination of multivariate Cox regression analysis was performed. Five genes linked to prognosis were discovered (Fig. 4A). Additional research, multivariate Cox regression analysis, and LASSO regression revealed that five genes (ABCA1, CD36, APOA1, APOC3 and NPC2) were prognostic markers that were used to construct prognostic models (Fig. 4B and 4C). First, five genes involved in cholesterol metabolism whose somatic mutation patterns are correlated with prognosis were examined. As seen in the figure (Fig. 4D), a total of 26 out of 431 GC samples had mutations in genes associated to cholesterol metabolism with a frequency of 6.03%. Risk scoring models were used for Principal Component Analysis (PCA) to distinguish between high-risk or low-risk GC samples (Supplementary Figure 1A and 1B).

### Clinical features and risk scores in relation

**Figure 5:**
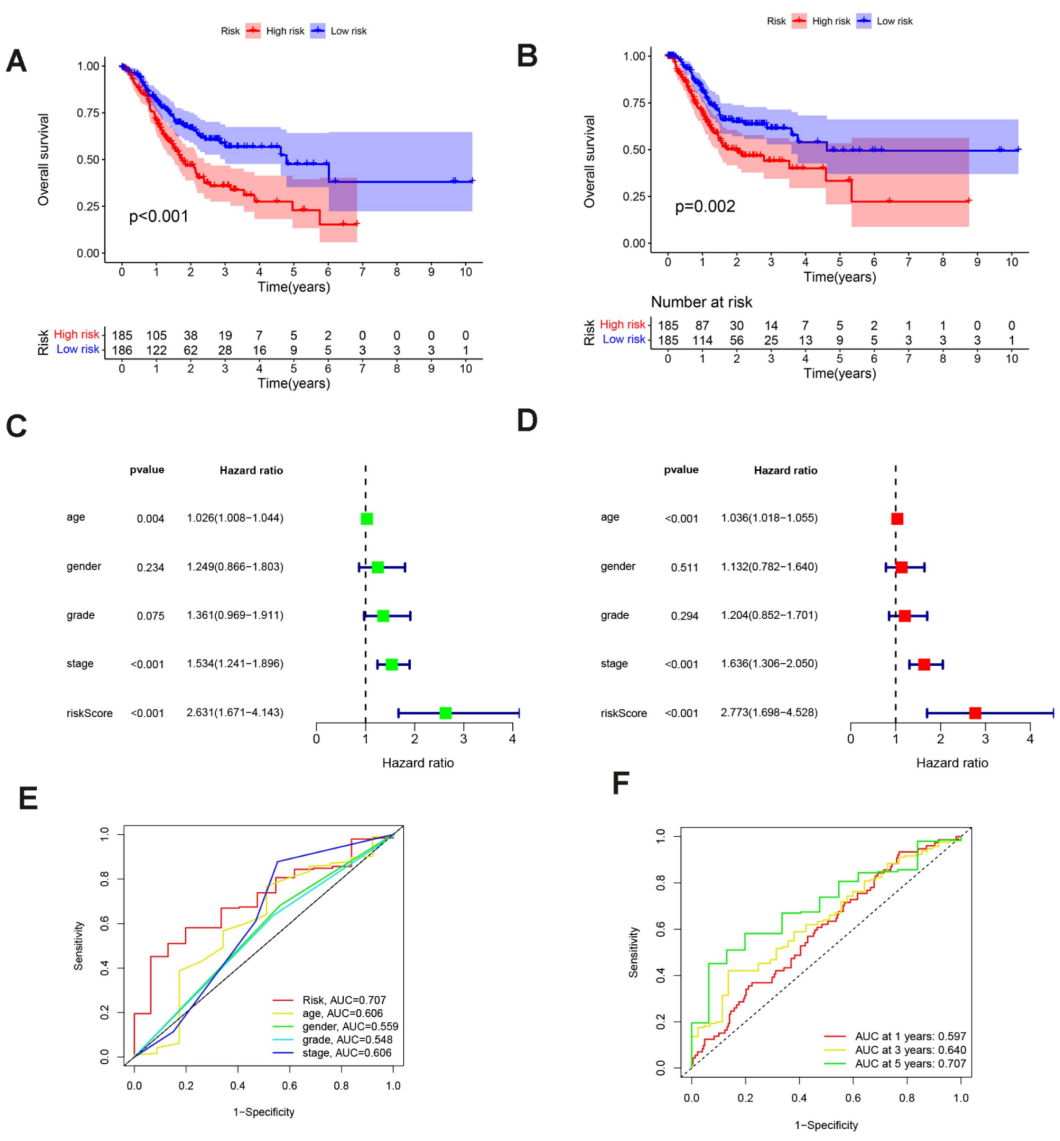
Evaluating Prognostic Value of Prognostic Risk Score Model: **(A, B)** Survival analysis between low- and high-risk groups in the training and validation cohorts. **(C, D)** Univariate and multivariate Cox regression forest plots for the TCGA cohort. **(E)** ROC curves for risk scores and clinical traits. **(F)** ROC curves of risk score models predicting patient survival.

The cutoff value was selected at the median of the risk scores in the training cohort. The sample was divided into groups with low (n = 186) and high (n = 185) risk scores using the cutoff values. Compared to the low risk score group, the sample in the high risk group had a worse outcome (p<0,001; Fig.5A). To confirm that the prognostic risk score model is accurate, samples from the GEO (GSE26899) were used as the validation cohort. The cutoff values determined from the training cohort were split into groups with high (n = 185) and low (n = 185) risk scores, resulting in a worse prognosis for the high risk score group sample (p = 0.002; Fig. 5B). This suggests that the overall survival (OS) in GC may be predicted by the prognostic risk score model. Univariate and multifactorial analyses showed that OS was independently predicted by risk score, age and clinical stage (Fig. 5C and 5D). The findings of the multivariate ROC analysis demonstrated that risk scores outperformed conventional pathological prognostic variables in predicting overall survival (Fig. 5E). In addition, the risk score model was highly accurate at forecasting patients' 5-year survival rates (Fig. 5F). We next analyzed the association of risk score grouping with the gastric cancer TNM stage categorization depending on the AJCC, pathologic stage, pathologic staging, and gender in the sample (Supplementary Figure 1C-1H). The findings revealed that increased risk scores were linked to increased AJCC-T (tumor invasion) (Supplementary Figure 1C), AJCC-N (lymphatic metastasis) (Supplementary Figure 1D), and pathological staging of gastric cancer (Supplementary Figure 1E).

### Making a nomogram to forecast a patient's prognosis for stomach cancer

**Figure 6:**
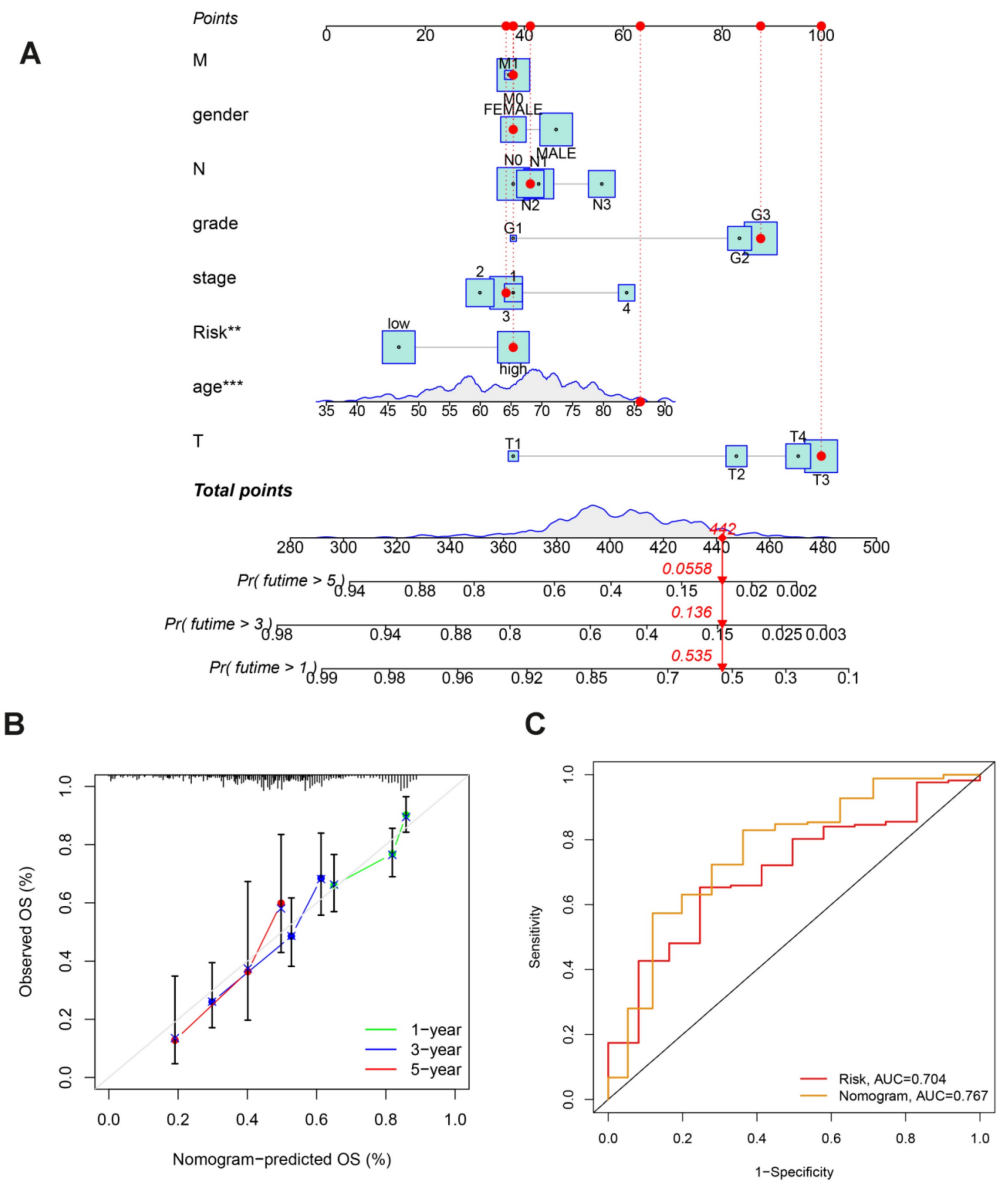
Nomogram Construction in TCGA Dataset: **(A)** Nomogram predicting OS in stomach cancer patients. **(B)** Calibration plot comparing predicted survival (x-axis) and actual survival (y-axis). **(C)** ROC curves comparing the performance of the nomogram and risk score.

In order to more accurately gauge GC patients' prognosis, a nomogram combining pathological stage, age, sex, pathological classification and prognostic risk score was constructed for the calibration curve demonstration of GC samples at 1, 3, and 5 years (Fig. 6A). Nomograms can accurately foresee the OS of people with GC (Fig. 6B). Furthermore, the nomogram performed better in predicting the prognosis of GC than the risk signature, as shown by the ROC curve (Fig. 6C). In conclusion, several factors were considered in evaluating our nomogram's effectiveness and dependability.

### Immune traits of clusters with low and high risk groups

**Figure 7:**
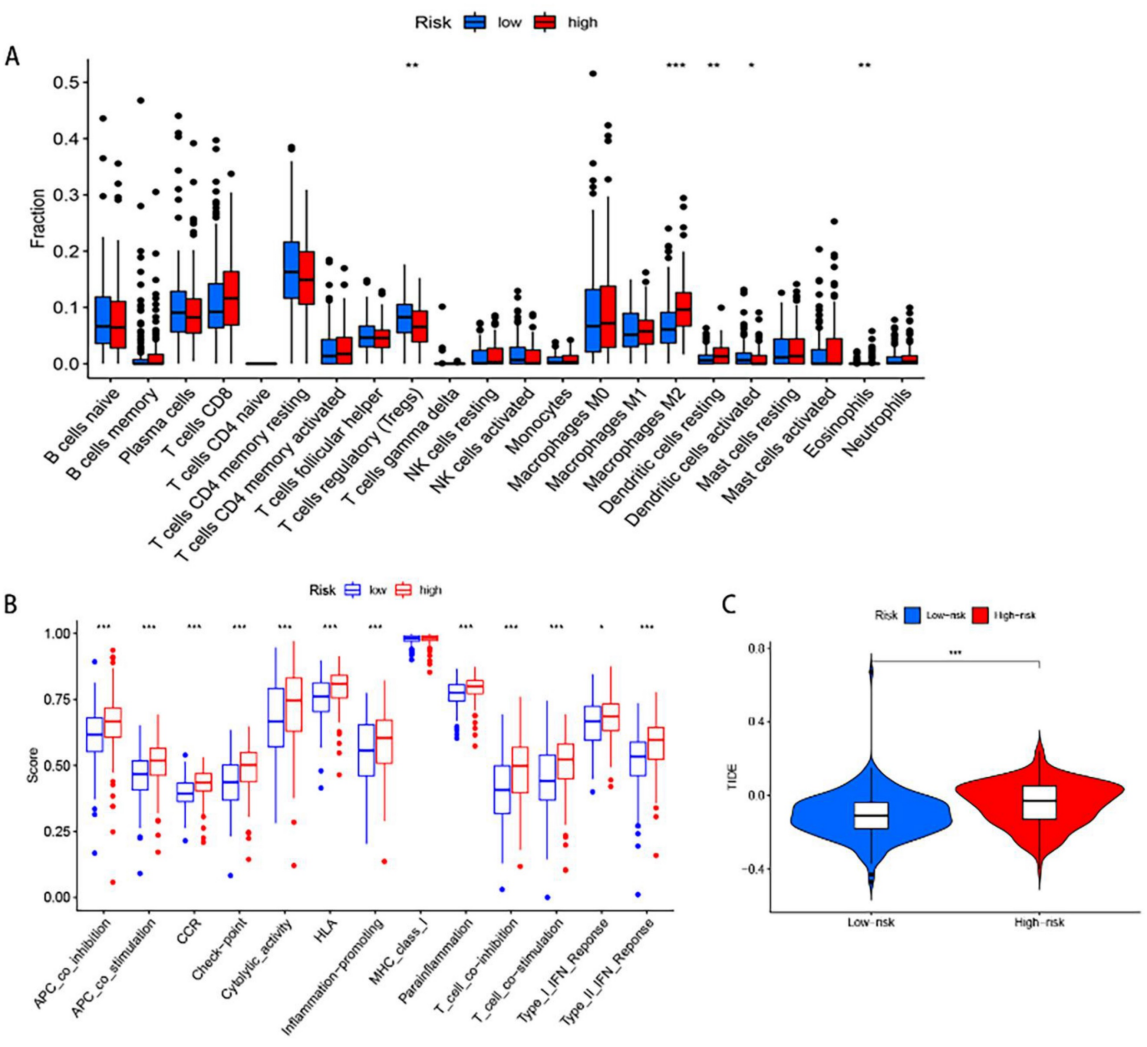
Immune Function Analysis: **(A)** Box plot showing differences in immune cell infiltration. **(B)** Box plot displaying differences in immune-related functional activity. **(C)** Violin plot illustrating immunotherapy response analysis.

To examine the connection between the risk score model and the immunological traits of people with GC, we first conducted a comparison of immune cells across the groups at high and low risk. The findings indicate that regulatory T cells (Tregs), M2 Macrophages, resting Dendritic cells, activated Dendritic cells and Eosinophils have a significant difference between the two groups (Fig.7A). Furthermore, immune-related functional analysis indicated that various immune functions such as APC co-inhibition, APC co-stimulation, CCR, check-point, cytolytic activity, HLA, inflammation-promoting, para-inflammation, T cell co-inhibition, T cell co-stimulation, type I interferon (IFN) response, and type II IFN response were activated in the high-risk group as shown in Figure 7B. Finally, the immunotherapy study revealed that high risk people had a higher probability of developing Tumor Immune Dysfunction and Exclusion (TIDE) and had less success with immunotherapy. This is consistent with poorer OS in the high- risk group.

### Expression verification of 5 differential genes in gastric cancer and normal tissues

**Figure 8:**
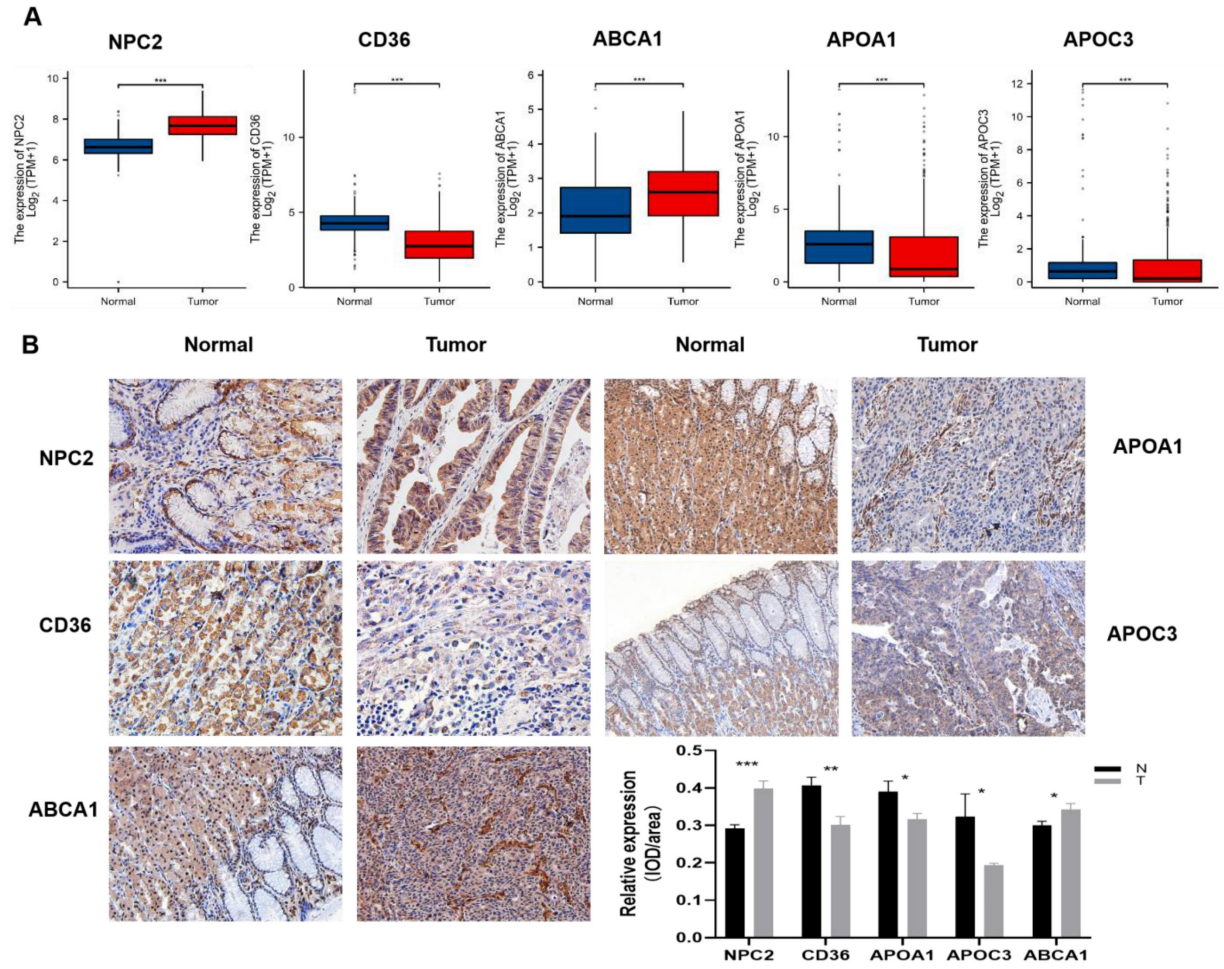
Differential Expression of Five Genes in Gastric Cancer: **(A)** TCGA and GTEx data show increased NPC2 and ABCA1 mRNA expression, and decreased APOA1, APOC3, and CD36 mRNA expression in gastric cancer. **(B)** Immunohistochemical staining of the five genes in clinical samples, with statistical analysis on the right.

Indeed, the TCGA and GTEx databases revealed that these five genes were differentially expressed in gastric cancer compared to the adjacent non-cancerous tissues, among which NPC2, ABCA1 were highly expressed in gastric cancer and APOA1, APOC3, CD36 were low expressed in gastric cancer (Fig.8A). To further validate these findings, we performed experimental verification. First, the protein expression of the five differentially expressed genes was evaluated by immunohistochemistry (Fig.8B), and the results were also as shown above. In addition, we also performed survival analysis of these five genes, and we found that except for the survival analysis of APOA1, patients with high expression of the other four genes (APOC3, NPC2, CD36, and ABCA1) had a poor prognosis (Supplementary Figure 2). We then examined the expression of these five genes in gastric cancer patient tissues and paired adjacent tissues by qPCR, and the same results were obtained (Supplementary Figure 3A). To further explore the validity of these 5 genes related to cholesterol metabolism, we selected one of the genes, NPC2, for *in vitro* validation. Western blot analysis was performed to assess the efficacy of the plasmid by transiently transfecting siNPC2 plasmid to investigate the role of NPC2 in gastric cancer (Supplementary Figure 3B).

### Knockdown of NPC2 significantly inhibits proliferation in GC

**Figure 9:**
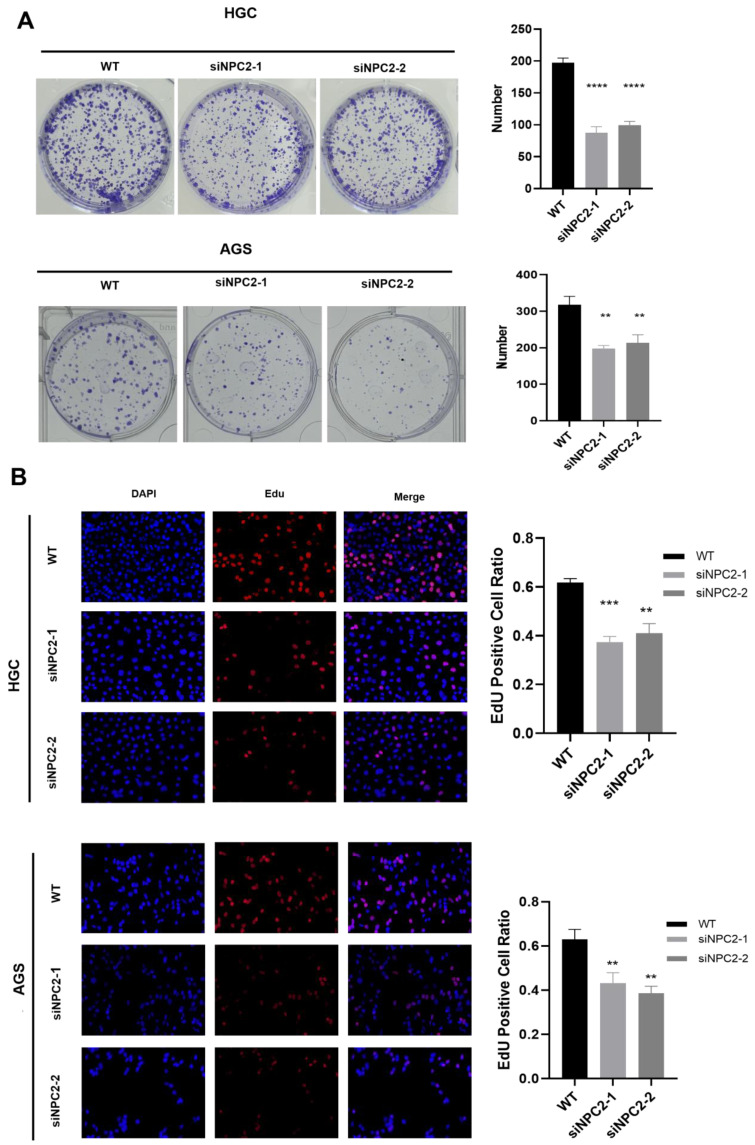
NPC2 promotes gastric cancer cell proliferation: **(A)** Cell colony formation assay confirms that knockdown of NPC2 affects the proliferation of gastric cancer cells **(B)** EdU assay was performed to assess cell proliferation. Red colour represents EdU-positive cells; blue color represents nuclei. The percentage of EdU-positive cells was determined by counting red/blue cells. *p< 0.05, **p< 0.01, ***p< 0.001.

We chose AGS and HGC cells for siRNA-mediated knockdown experiments to study the role of NPC2 in GC. The effectiveness of NPC2-siRNA was confirmed using western blot analysis (Supplementary Figure 4A). Subsequently, we carried out a number of experiments. NPC2 knockdown dramatically reduced the viability of HGC and AGS cells, as demonstrated by the CCK-8 experiment (Supplementary Figure 4B). Furthermore, NPC2 knockdown significantly decreased the number of HGC and AGS cell colonies, according to the cell colony formation assay (Fig. 9A). Lastly, the proliferative potential of HGC and AGS cells was assessed using the EdU method (Fig.9B). The findings indicated that there were less positive cells in the NPC2 knockdown group than in the normal group. To sum up, NPC2 may encourage the growth of stomach cancer cells.

### NPC2 promotes EMT in gastric cancer cells

**Figure 10:**
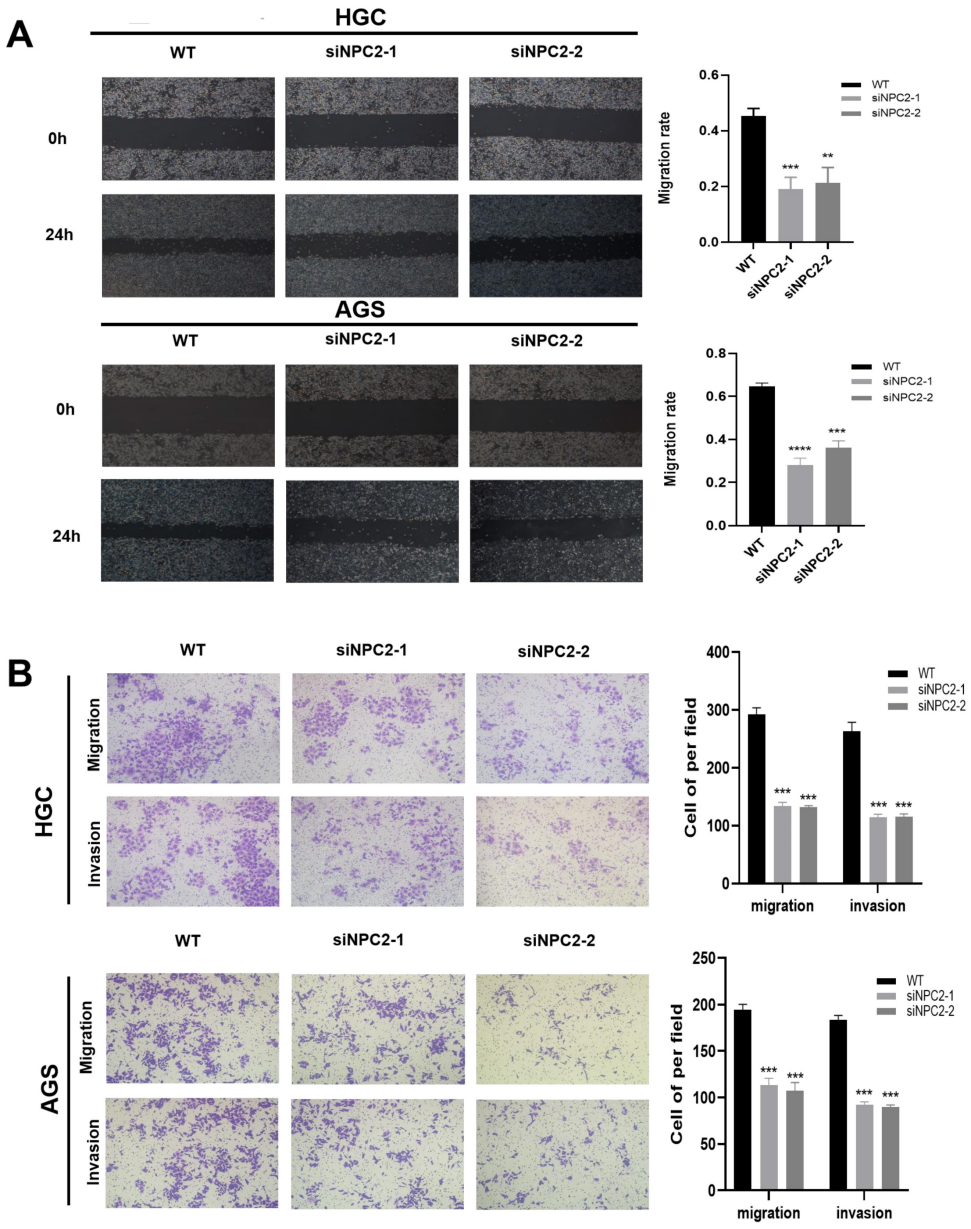
NPC2 promotes EMT in gastric cancer cells: **(A)** Wound healing assay was performed to assess cell migration ability. **(B)** Transwell assay was performed to evaluate cell migration and invasion ability.

We theorized that NPC2 could impact the course of epithelial-mesenchymal transition (EMT) in gastric cancer cells due to the importance of cholesterol metabolism-related genes in tumor migration and invasion in oncology research. First, we used a wound healing assay to examine the stomach cancer cells' ability to migrate. As Figure 10A illustrates, wound healing was sluggish in the NPC2 knockdown group. In the meantime, NPC2 knockdown decreased HGC and AGS cells' capacity for invasion and migration, according to the Transwell Assay (Fig. 10B). Lastly, we carried some WB tests to investigate the connection between NPC2 and EMT in more detail. The findings further supported the outcomes of the functional tests previously mentioned, demonstrating that downregulating NPC2 decreased the amounts of proteins in HGC and AGS cells that are linked to the EMT pathway, including E-cadherin, N-cadherin, β-catenin, Snail, and vimentin (Supplementary Figure 4). Overall, these findings indicate that NPC2 enhances the movement and penetration of gastric cancer cells.

## Discussion

In the past, one of the most prevalent malignancies in the globe was gastric cancer (GC). Among these, cholesterol metabolism plays a significant part in tumors 21. Cholesterol is an important component of biological membranes and serves as a signaling molecule and an important source of energy 22. In our study, Using bioinformatics analysis, the probable mechanism and prognostic significance of genes related to cholesterol metabolism in GC were thoroughly examined. By comparing the differentially expressed genes in the TCGA dataset between stomach cancer and healthy gastric epithelial tissues, 28 genes related to cholesterol metabolism and with significant differential expression were obtained. Notably, a predictive risk score model was developed using differential expression of five genes related to cholesterol metabolism in normal and cancer samples, which was applied to predict OS in patients with GC. Between the group of GC individuals with low risk and high risks scores, there was a difference in survival. The risk score model may be used to recognize individuals with poor survival, according to the results.

To enhance comprehension of the prognostic risk score model in GC, the clinical attributes of individuals in both the low-risk and high-risk score groups were juxtaposed. Elevated risk scores in GC patients correlated with diminished OS, implying that the cholesterol prognostic risk score model could be employed to customize treatments for individuals with GC.

The evidence indicates that tamoxifen has the potential to promote oncogenesis by inhibiting cholesterol transport in lysosomes and cholesterol synthesis 23. Nevertheless, the regulation of cholesterol metabolism in tumour cells through the use of antimetabolic drugs alone has been demonstrated to be an ineffective method for tumour reduction. However, it has been shown to have the potential to alter the tumour microenvironment and enhance the efficacy of other therapeutic agents. The combination of lovastatin and tamoxifen has been demonstrated to markedly enhance the antitumour efficacy of tamoxifen 24. The administration of Avamab was observed to enhance the immune function of CD8+ T cells, as evidenced by an increase in cholesterol concentration within these cells. Additionally, the combination of Avamab and a PD-1 monoclonal antibody demonstrated promising anti-tumour synergy. It is therefore imperative that cholesterol metabolism drugs are targeted in combination with other targeted therapies, as well as immunotherapy 25.

So, it was determined whether there was a connection between the risk score and immune cell infiltration. The findings revealed that the high risk group had considerably more M2 Macrophages cells than the low risk group. Macrophages in M2 can promote tumor progression and reduce the benefit of immunotherapy by suppressing effective anti-tumor immunity. APC co-inhibition, APC co-stimulation, CCR, check-point, cytolytic activity, HLA, inflammation-promoting, parainflammation, T cell co-inhibition, T cell co-stimulation, and type II IFN expression were all higher in the high risk group of GC patients than they were in the low risk group, suggesting that patients in the high risk group had a poor prognosis, which was partly brought on by the immunosuppressive environment and increased immune checkpoint epigenetics 26. In addition, our findings suggest that it is crucial to identify patients who are eligible for immunotherapy in clinical practise because individuals in the high risk group have a higher likelihood of developing TIDE.

Previous studies have shown that genes involved in cholesterol metabolism are associated with cancer progression and have either favorable or unfavorable prognostic significance in various tumors 27. High ABCA1 expression promotes a poor immune microenvironment and low survival in patients with gastric adenocarcinoma 28, and APOA1 serves as a novel therapeutic option to inhibit colorectal cancer-promoting metastasis by modulating intracellular cholesterol metabolism 29. Notably, NPC2 is a secreted glycoprotein that plays an important role in the regulation of intracellular free cholesterol homeostasis. Patients with NPC2 downregulation express higher alpha-fetoprotein, a wide range of tumor types, vascular infiltration, later pathological stage, and lower survival 30. In patients with hepatocellular carcinoma, NPC2 downregulation is associated with adverse clinicopathological features by regulating mitogen-activated protein kinase (MAPK)/extracellular signal-regulated kinase (ERK) activation 31. And NPC2 inhibits maladaptive tissue remodeling and inflammatory development by negatively regulating ERK 1/2 MAPK phosphorylation 32. In addition, low expression of NPC2 in hepatocellular carcinoma tissues may indicate poor postoperative prognosis in hepatocellular carcinoma patients 33. Another study showed that cholesterol-binding protein NPC2 inhibits the recruitment of stromal macrophages to early lung tumors 34. NPC2 is a potential therapeutic target for the treatment of type 2 diabetes mellitus and related metabolic disorders 35. These suggest that these five cholesterol-related genes (APOA1, APOC3, ABCA1, NPC2, CD36) play an important role in cancer. There have been many studies related to NPC2 in hepatocellular carcinoma, but very few studies have been conducted in gastric cancer. Therefore, to clarify NPC2's role in gastric cancer and evaluate its potential as a therapeutic target, bioinformatics analysis and experimental validation were carried out around NPC2 in this work. According to our findings, NPC2 expression was significantly upregulated in gastric cancer tissues and cell lines, and downregulation of NPC2 expression significantly reduced the ability of gastric cancer cells to proliferate, invade, migrate, and undergo epithelial-mesenchymal transition (EMT). Thus, we suggest that therapeutic targeting of NPC2 may enhance the prognosis for patients with gastric cancer and promote additional research to identify the precise mechanisms by which NPC2 regulates the development of GC.

Our study has some limitations. Initially, we validated the precision of our model utilizing TCGA and GEO data sets, lacking substantial reliance on our exclusive clinical samples for authentication. Furthermore, with regards to NPC2, our investigations were restricted to *in vivo* and *in vitro* assessments, omitting animal trials for validation. Lastly, our focus was solely on illustrating alterations in the expression of select epithelial-mesenchymal transition (EMT)-related proteins, forgoing an in-depth exploration of downstream mechanisms. Moving forward, additional researchers are essential for corroborating these findings and unveiling novel molecular targets crucial for gastric cancer treatment.

## Conclusion

In summary, this paper investigated the expression and function of genes related to cholesterol metabolism in GC using a comprehensive bioinformatics analysis. A novel prognostic risk scoring model containing five genes for cholesterol metabolism was developed and validated for the comprehensive assessment of outcomes in patients with gastric cancer. It was found that NPC2 is a significant factor in gastric cancer and serves as a key prognostic factor. Additionally, the study demonstrated that NPC2 promotes the progression of gastric cancer and the development of EMT. These findings offer new potential targets for predicting prognosis and treatment of gastric cancer.

## Supplementary Material

Supplementary figures and table.
