# Supplementary material for: Cholesterol metabolism-related genes predict immune infiltration and prognosis in gastric cancer patients
Source: J Cancer. 2025 Mar 10;16(7):2087–102. doi: 10.7150/jca.104389 (PMC12036097; doi:10.7150/jca.104389)

Table S1 50 genes related to cholesterol metabolism

CYP27A1	CYP7A1	SOAT	LCAT	LIPA	LPL	APOE	LRP1	ABCA1	ABCB11
ABCG5	ABCG8	TSPO	VDAC1	VAPA	LRP2	CD36	APOA1	APOA2	APOC3
APOA4	PLTP	ANGPTL4	LPA	MYLIP	VAPB	NPC1	SORT1	LDLR	LDLRAP1
PCSK9	NPC2	SCARB1	NCEH1	APOB	VDAC2	VDAC3	CETP	STAR	APOH
OSBPL5	LIPC	LIPG	APOC1	APOC2	ANGPTL3	ANGPTL8	LRPAP1	STARD3	CIDEB

Supplementary Figure 1 Pre-modeling of genes involved in cholesterol metabolism:
(A) Cholesterol metabolism-related genes Uncorrected pre-PCA
(B) Cholesterol metabolism-related genes corrected PCA
(C-H) Association between risk scores and clinicopathological features, including tumor invasion (C), lymphatic metastasis (D), distant metastasis (E), TNM stage (F), pathological type (G), and gender (H).

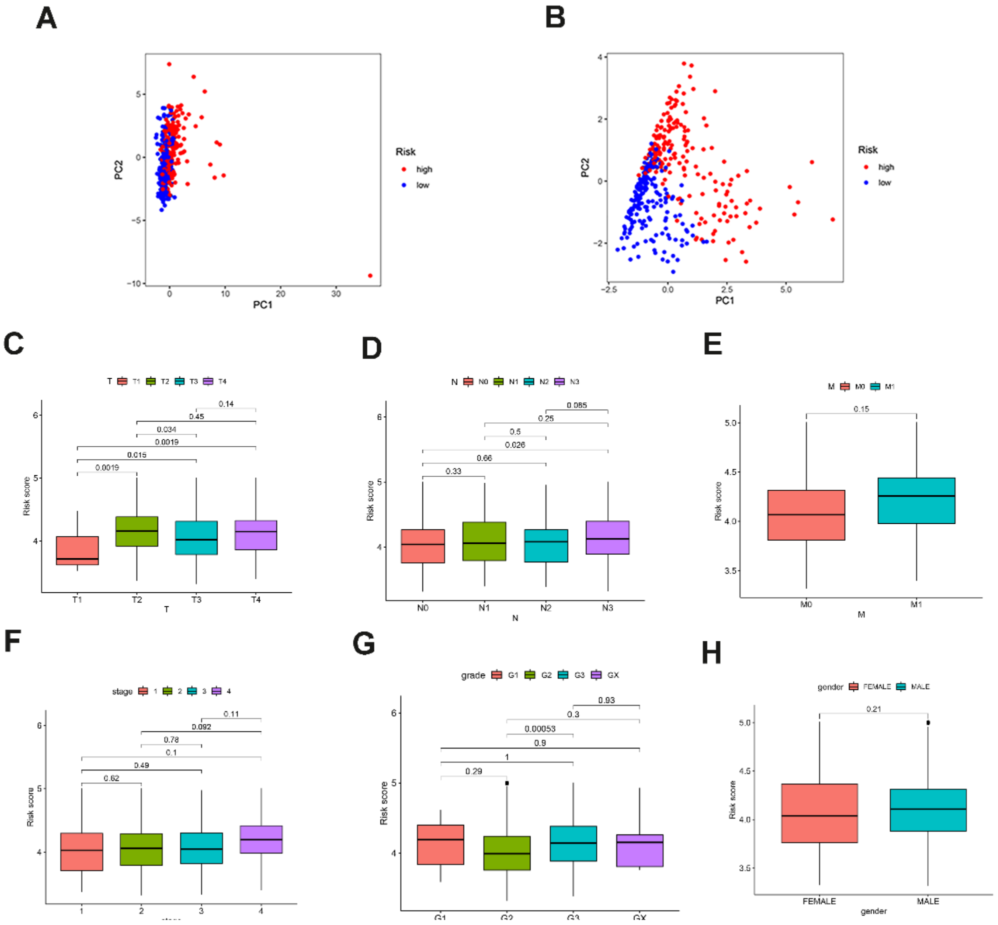

Supplementary Figure 2 Survival analysis of five genes in gastric cancer patients.

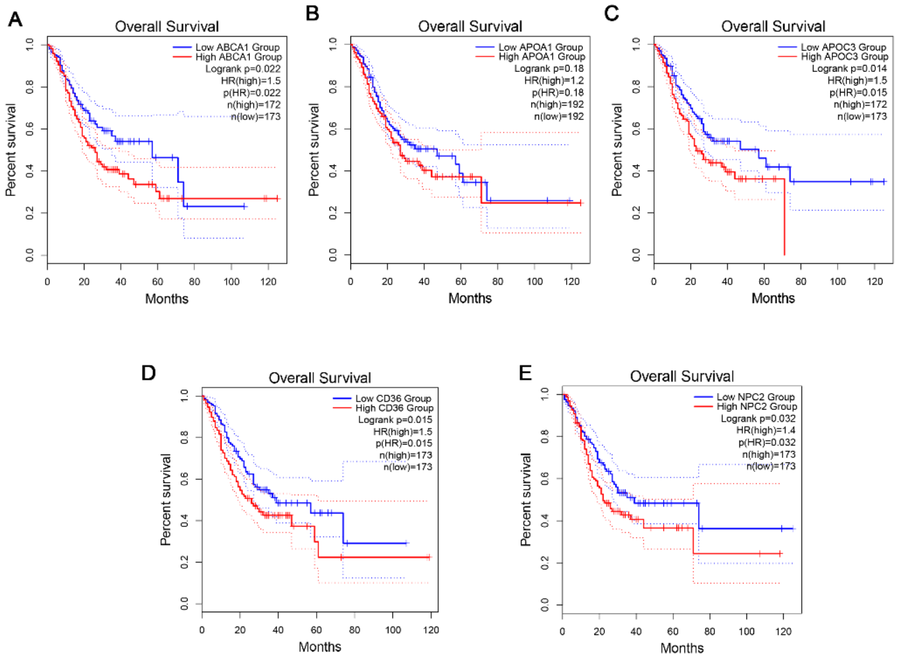

Supplementary Figure 3 Validation of NPC2 knockdown in gastric cancer cells:

(A)Western blot validation of NPC2 overexpression in gastric cancer cells.

(B) Knockdown of NPC2 reduced the number of colonies in HGC and AGS cells.

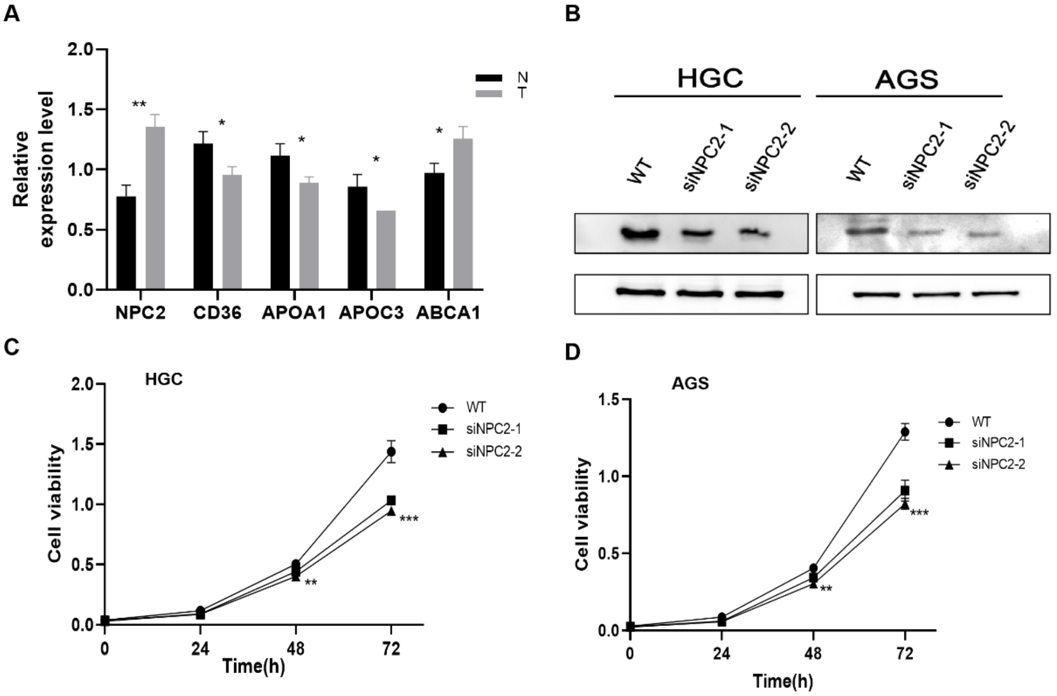

Supplementary Figure 4 Changes in E-cadherin, N-cadherin, β-catenin, snail and vimentin proteins after Western blot of NPC2 downregulation. *p<0.05, **p<0.01, ***p<0.001.

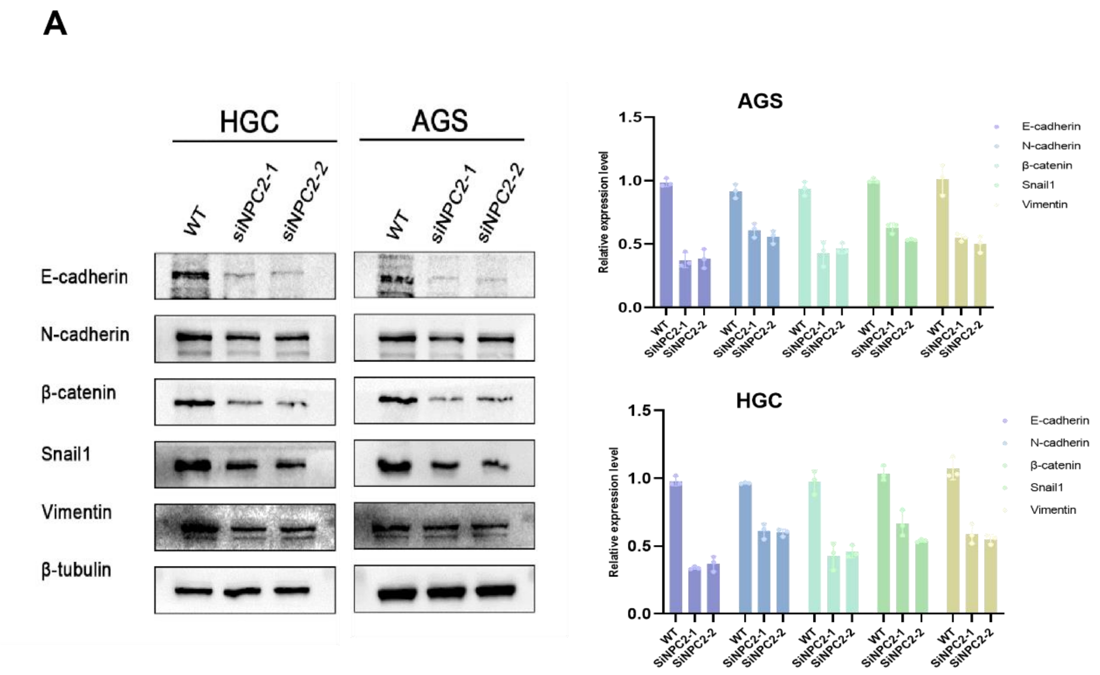

Supplement: Supplementary file 1 — Supplementary figures and table. [file jcav16p2087s1.pdf]
